# Nocturnal Cardiac Arrhythmias in Sleep Apnoea After Acute Myocardial Infarction and the Effect of Adaptive Servo-Ventilation: An Ancillary Study of the TEAM-ASV I Trial

**DOI:** 10.3390/jcdd13040157

**Published:** 2026-04-02

**Authors:** Jan Pec, Marek Nigl, Henrik Fox, Stefan Stadler, Michael Kohn, Sarah Driendl, Olaf Oldenburg, Florian Zeman, Stefan Buchner, Michael Arzt

**Affiliations:** 1 Department of Internal Medicine II, University Hospital Regensburg, Franz-Josef-Strauss-Allee 11, 93053 Regensburg, Germany; 2 Clinic for General and Interventional Cardiology/Angiology, Heart and Diabetes Center, NRW Ruhr University Bochum, 32545 Bad Oeynhausen, Germany; 3 Center for Cardiology, Ludgerus-Kliniken, 48153 Münster, Germany; 4 Center for Clinical Studies, University Hospital Regensburg, 93053 Regensburg, Germany; 5 Internal Medicine II—Cardiology, Sana Clinics of the District of Cham, 93413 Cham, Germany

**Keywords:** adaptive servo-ventilation, acute myocardial infarction, sleep-disordered breathing, cardiac arrhythmias

## Abstract

(1) Background: Early treatment of sleep-disordered breathing (SDB) with adaptive servo-ventilation (ASV) after acute myocardial infarction (AMI) has been shown to improve myocardial salvage. This analysis evaluates nocturnal electrocardiogram (ECG) Holter data, derived from polygraphy in a randomised clinical trial (NCT02093377), to assess the occurrence of nocturnal cardiac arrhythmias in patients with SDB and to explore the effect of ASV therapy. (2) Methods: In the TEAM-ASV I trial, patients were stratified by the presence/absence of SDB, defined by an apnoea–hypopnoea index (AHI) ≥15 events/h assessed with polygraphy. Those with SDB were subsequently randomised to receive ASV in addition to standard AMI care. Guideline-conforming semi-automated ECG analysis of nocturnal cardiac arrhythmias was conducted via Holter–ECG software (custo diagnostic, version 5.4). (3) Results: Patients with SDB had an increased incidence of non-sustained ventricular tachycardia (NSVT) (SDB: n = 8 (16%) vs. no SDB: n = 1 (2%); *p* = 0.024) and premature atrial contractions (PAC) (SDB: 1.2/h [0.3, 3.4] vs. no SDB: 0.3/h [0.1, 1.2]; *p* = 0.017). In patients with SDB who were randomised to ASV treatment early after AMI, we found no reduction in cardiac arrhythmias when ASV was added to standard care. (4) Conclusions: After AMI, SDB was linked to increased NSVT and PAC. ASV treatment demonstrated neither a harmful nor a beneficial effect on the occurrence of nocturnal cardiac arrhythmias. Further trials are warranted to confirm these findings.

## 1. Introduction

Sleep-disordered breathing (SDB) is highly prevalent in patients with cardiovascular disease and particularly common after acute myocardial infarction (AMI) [1,2]. Growing evidence suggests that SDB contributes to cardiac arrhythmogenesis and may represent an important risk factor for adverse cardiac outcomes, including recurrent ischemic events, heart failure, and mortality [3,4,5,6,7,8]. One of the mechanisms linking SDB to adverse cardiac outcomes is the imbalance between myocardial oxygen supply and demand during apnoeic events. Recurrent episodes of hypoxaemia occur simultaneously with acute increases in intrathoracic pressure, systemic arterial pressure, and sympathetic activation, which together raise myocardial oxygen demand while oxygen delivery is reduced. This mismatch may render the myocardium particularly vulnerable to nocturnal ischemia, reinfarction, and ventricular arrhythmias [9]. In addition, nocturnal hypoxaemia occurring in SDB was associated with larger infarct size after AMI [10].

The early identification and treatment of SDB in the acute phase after AMI may therefore have therapeutic relevance. The TEAM-ASV I randomised trial demonstrated that early treatment with adaptive servo-ventilation (ASV) after AMI significantly improved myocardial salvage index and reduced infarct size, suggesting a beneficial effect of treating SDB on myocardial recovery [11]. However, the impact of ASV on nocturnal cardiac arrhythmias in the early post-infarction period remains insufficiently characterised. Given that arrhythmias are common after AMI and are associated with worse prognosis, understanding whether the treatment of SDB can modulate arrhythmic burden may have important clinical implications.

Therefore, the aim of this ancillary analysis was to evaluate whether nocturnal cardiac arrhythmias are more prevalent in patients with SDB following AMI and to investigate whether treatment with ASV is associated with a reduction in arrhythmic events.

## 2. Methods

In this ancillary analysis of the investigator-initiated, parallel, open-label, proof-of-concept randomised controlled TEAM-ASV I trial (NCT02093377) with patient recruitment from February 2014 to August 2020, we analysed existing polygraphy data at baseline and at 12-week follow-up. All patients were admitted to one of three hospitals in Germany with a first AMI with ST elevation or acute occlusion of a coronary artery and had undergone primarily successful percutaneous coronary intervention within 24 h of symptom onset. The eligibility criteria have been previously reported in detail [12]. Data on demographic and clinical variables were collected prospectively after enrolment within the framework of this randomised prospective study.

For the cross-sectional analysis, we additionally included enrolled patients without SDB (apnoea–hypopnoea index < 15 events·h^−1^) who were not part of the main trial population, restricted to patients with SDB and for whom only baseline data were available. Patients with SDB were randomly assigned to either the ASV group, receiving ASV therapy (AutoSet CS–PaceWave, ResMed Corp., San Diego, CA, USA) in addition to standard care of AMI, or the control group, receiving standard care only. Randomisation was conducted in blocks of six using an online tool (www.randomizer.at), allocating participants to the ASV or control group in a 1:1 ratio. Stratification factors included infarct location (LAD vs. other culprit lesions) and study site. Longitudinal follow-up analyses were performed exclusively in randomised patients with SDB.

All patients provided written informed consent. The trial was approved by the institutional ethics committee of University Hospital Regensburg, Regensburg, Germany (approval number 14-122-0031, approval date 23 January 2014) and conducted in accordance with the Declaration of Helsinki.

ECG signal was derived from overnight polygraphy (SOMNOscreenTM plus RC, SOMNOmedics, Randersacker, Germany) performed within 3 days after PCI and at the 12-week follow-up visit. Polygraphy data was transferred to the Centre of Sleep Medicine at University Hospital Regensburg. After extraction of the nocturnal electrocardiogram (ECG) Holter data of up to three precordial leads with sampling frequency of 256 Hz using EDF + data format, two blinded investigators assessed the quality of the signal. Semi-automated analysis was conducted using commercially available software (custo diagnostic, version 5.4, custo med GmbH, Ottobrunn, Germany). Both investigators scored nocturnal cardiac arrhythmia using guideline-conforming definitions. Details on quality assessment and scoring were published previously in detail [13]. Non-sustained ventricular tachycardia (NSVT) was defined as ≥3 consecutive ventricular beats lasting <30 s.

Descriptive data expressed as means ± SD, medians with IQR, or frequencies (%) were compared using the unpaired *t*-test, Mann–Whitney U test, or chi-squared test, respectively. The sample size of this ancillary study was restricted to available polygraphy data suitable for the automated analysis and not to a prospective effect size calculation. Two-sided *p* ≤ 0.05 was defined as statistically significant. Statistical analysis was performed in SPSS (SPSS Statistics for macOS, Version 29.0, Armonk, NY, USA: IBM Corp).

## 3. Results

Of the 171 patients tested for eligibility, 79 were diagnosed with SDB and were randomised to either ASV treatment or a control group (Figure S1). A total of 74 patients had no SDB. Of the 104 original nocturnal ECG recordings from polygraphy, 94 were analysable based on technical requirements and quality (Figure S1). Nocturnal ECG Holter recordings at baseline and 12-week follow-up were available for 32 patients with SDB randomised to ASV treatment or to the control arm. Other reasons for loss to follow-up were reported previously [11]. The mean ECG recording duration was 8.3 ± 1.3 h for baseline and 8.2 ± 1.1 h for follow-up. Patient baseline characteristics are shown in Table 1 for the cross-sectional analysis and in Table S1 for the longitudinal analysis. Patients with SDB were significantly older, more often male, and more likely to receive spironolactone compared to those without SDB. Both the ASV and control groups exhibited severe SDB, but the apnoea index was higher in the ASV group (Table S1). Both SDB groups were dominated by obstructive sleep apnoea, albeit with a relatively high proportion of central events (Table S1). Other baseline characteristics were similar for both groups (Table S1). The safety profile of the intervention has been previously reported, with no serious treatment-related adverse events observed during the study [11].

### 3.1. Sleep-Disordered Breathing and Nocturnal Cardiac Arrhythmia

**Figure 1:**
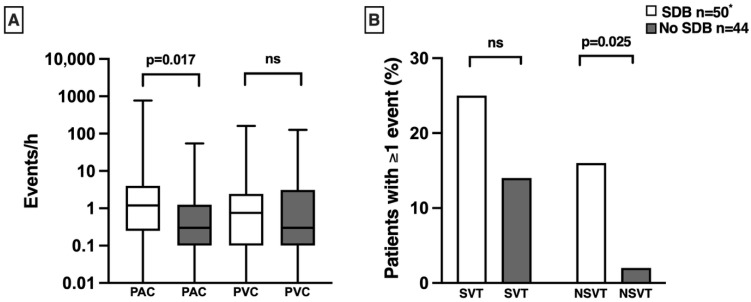
Comparison of nocturnal cardiac arrhythmias between subjects with and without sleep-disordered breathing (SDB): complete case analysis (n = 94). (**A**) Median number of premature ventricular contractions (PVCs) and premature atrial contractions (PACs) per hour, along with interquartile ranges. Patients with SDB exhibit a higher frequency of PACs compared to those without SDB (1.2 [0.3, 3.4] vs. 0.3 [0.1, 1.2], *p* = 0.017). (**B**) Proportion of patients experiencing more than one episode of non-sustained ventricular tachycardia (NSVT) and supraventricular tachycardia (SVT) per night, presented as percentages. Notably, NSVT is more common among patients with SDB compared to those without (16% vs. 2%, *p* = 0.025). *p*-values derived from Mann–Whitney U test or chi-squared test. A logarithmic scale was used for the *Y*-axis in panel (**A**). ns: not significant. * One patient in the SDB group was excluded from the analysis of PAC and SVT due to the presence of atrial fibrillation.

Non-sustained ventricular tachycardias (NSVTs) were more prevalent in patients with SDB (Table 1, Figure 1). However, the number of premature ventricular ectopic beats (PVCs) was similar between both groups (Table 1, Figure 1). For supraventricular arrhythmias, the number of ectopic beats (PACs) was higher in patients with SDB. Supraventricular tachycardias (SVTs) were numerically more frequent in patients with SDB (Table 1, Figure 1). We found no difference between both groups for couplets, bigeminy, or trigeminy (Table 1). No sustained ventricular tachycardias were observed. One patient in the SDB group without follow-up ECG had atrial fibrillation as a predominant cardiac rhythm and was excluded from the analysis of PAC and SVT.

### 3.2. Effect of Adaptive Servo-Ventilation on Nocturnal Cardiac Arrhythmia

**Figure 2:**
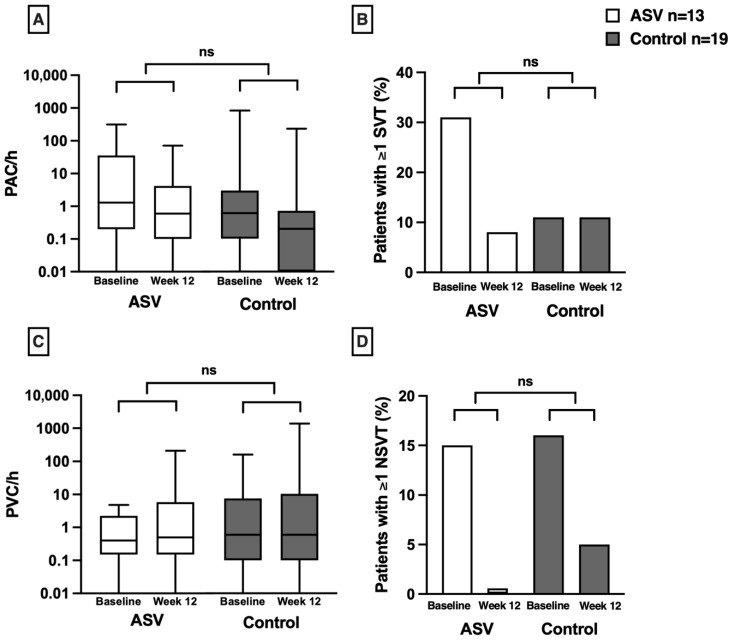
Nocturnal cardiac arrhythmias in patients with sleep-disordered breathing (SDB) at baseline and follow-up at week 12, comparing those randomised to adaptive servo-ventilation (ASV) treatment (white) versus control group (grey): subgroup analysis (n = 32). The upper panels depict supraventricular arrhythmias: (**A**) the number of premature atrial contractions (PACs) per hour and (**B**) the percentage of patients with more than one episode of supraventricular tachycardia (SVT) per night. The lower panels show ventricular arrhythmias, showing (**C**) the number of premature ventricular contractions (PVCs) per hour and (**D**) the percentage of patients with more than one episode of non-sustained ventricular tachycardia (NSVT) per night. *p*-values derived from Mann–Whitney U test or chi-squared test. A logarithmic scale was used for the *Y*-axis in panels (**A**,**C**). ns: not significant.

No clinically significant difference was observed for supraventricular or ventricular arrhythmia events across both groups (Table 2, Figure 2). A decline in the number of NSVTs over 12 weeks was present in both groups (Table 2, Figure 2). PVCs, PACs, SVT, couplets, bigeminy, and trigeminy showed no clinically relevant decrease (Table 2, Figure 2). No patient developed atrial fibrillation or flutter during follow-up.

## 4. Discussion

This study offers new insights into the occurrence of nocturnal cardiac arrhythmias in patients with SDB and the effect of ASV treatment early after AMI. First, a significantly greater number of NSVT episodes was observed in patients with SDB, although the absolute number of events was small. Second, the addition of ASV to standard post-AMI care did not significantly influence atrial and ventricular ectopic activity or the incidence of nocturnal atrial and ventricular tachycardia.

The incidence of ventricular tachycardia after AMI is a strong predictor of mortality [14,15]. In this study, we show that SDB is associated with a higher prevalence of NSVT compared to patients with no SDB. Following myocardial infarction, the arrhythmogenic substrate is characterised by ischemic injury, scar formation, and autonomic imbalance. Superimposed intermittent hypoxia, intrathoracic pressure alterations, and sympathetic surges associated with SDB may further enhance ventricular electrical instability [3]. SDB is associated with greater infarct size and impaired cardiac healing after AMI [16], which may also contribute to arrhythmogenesis. The clinical relevance of PVC burden in our study population remains uncertain, as no universally accepted post-AMI threshold has been established in the contemporary PCI era, and PVC-related risk depends strongly on the overall arrhythmic substrate [17,18]. Notably, PVC burden in our study was low across all groups and below levels historically associated with adverse prognosis after myocardial infarction [17,18].

To our knowledge, the nocturnal ECG Holter recordings from patients randomised to ASV treatment were analysed only in the SERVE-HF trial [19]. The authors concluded no significant effect of ASV treatment on nocturnal ventricular ectopy or tachyarrhythmia over a period of 12 months. However, this population differs substantially as the patients in SERVE-HF trial exclusively had central sleep apnoea and chronic heart failure with reduced ejection fraction, and had no recent AMI. In the SERVE-HF trial, ASV had no effect on the primary composite endpoint and was associated with significantly higher all-cause and cardiovascular mortality [20]. By contrast, in ADVENT-HF, the use of a newer-generation, peak-flow triggered ASV device without fixed pressure support was not associated with a safety signal; although it did not improve the primary composite outcome or mortality, it showed positive effects on patient-reported outcomes [21]. Recent American clinical guidance and the joint European Respiratory Society/European Sleep Research Society position paper support a more differentiated reassessment of ASV therapy in patients with preserved ejection fraction and selected patients with reduced ejection fraction in the range of 30–45% [22,23]. Another small, randomised trial in patients with heart failure with reduced ejection fraction and obstructive sleep apnoea receiving continuous positive airway pressure suggested a decrease in PVCs paralleled by a reduction in urinary norepinephrine concentrations [24].

Based on findings from our previous observational study [1,2], ASV was chosen over continuous positive airway pressure (CPAP) because central sleep apnoea was highly prevalent in this population. While CPAP is an established treatment for obstructive sleep apnoea, ASV was considered more appropriate in the present setting because it can more effectively address both obstructive and central respiratory events.

A potential limitation of our study is that we may have missed earlier effects of ASV therapy, as a previous study reported improvements in cardiac repolarization at 4 weeks that were not sustained at 12 weeks [25]. Importantly, prolonged cardiac repolarization is considered a surrogate marker for malignant ventricular arrhythmias and sudden cardiac death [26,27]. Moreover, our analysis of Holter ECG recordings was limited to overnight monitoring. Additional daytime monitoring would allow us to explore the effects of ASV treatment beyond the nocturnal phase and provide further information on associated symptoms. Furthermore, we used polygraphy rather than polysomnography for the diagnosis and monitoring of ASV treatment, which is not the standard method and therefore may have limited accuracy. Finally, the final sample size fell below the calculated target due to issues attributable to the COVID-19 pandemic and slow recruitment at the beginning of the study. Especially, the limited sample size of the randomised subgroup with longitudinal follow-up data substantially restricts statistical power. Therefore, the absence of significant treatment effects should be interpreted with caution.

In summary, we demonstrated that, in patients after AMI, SDB was associated with a higher occurrence of NSVT and PAC. Moreover, in patients with SDB who were randomised to ASV treatment early after AMI, we found no reduction in cardiac arrhythmias when ASV was added to standard care. Our data suggest that early ASV treatment neither triggers nor reduces the occurrence of nocturnal atrial and ventricular arrhythmias. These findings should be regarded as exploratory and require confirmation in larger randomised trials.

## Figures and Tables

**Table 1:** Demographics, clinical characteristics, and nocturnal ECG Holter findings at baseline.

	Total (n = 94)	SDB (n = 50)	No SDB (n = 44)	*p*-Value
Age, years	56 ± 11	60 ± 11	52 ± 10	<0.001 ^T^
Body mass index, kg/m^2^	29.4 ± 5.1	30.2 ± 6.2	28.5 ± 3.6	0.129 ^T^
Male sex	50 (53%)	40 (80%)	10 (23%)	<0.001 ^Chi^
Hypertension	59 (63%)	31 (62%)	28 (64%)	0.870 ^Chi^
Current smoker	49 (53%)	25 (50%)	24 (56%)	0.576 ^Chi^
Diabetes mellitus	11 (12%)	7 (14%)	4 (9%)	0.460 ^Chi^
Pain-to-balloon time, h	3.6 [2.7, 6.1]	3.8 [2.7, 6.1]	3.5 [2.7, 6.1]	0.443 ^MWU^
Left ventricular ejection fraction (%)	53 ± 9	51 ± 10	54 ± 7	0.097 ^T^
NSTEMI	8 (9%)	5 (10%)	3 (7%)	0.561 ^Chi^
Culprit lesion				
Left main artery	4 (4%)	1 (2%)	3 (7%)	0.248 ^Chi^
LAD artery	64 (68%)	35 (70%)	29 (66%)	0.671 ^Chi^
Circumflex artery	32 (34%)	14 (28%)	18 (41%)	0.188 ^Chi^
Right coronary artery	46 (49%)	24 (48%)	22 (50%)	0.847 ^Chi^
Coronary flow before PCI: TIMI 0–1	82 (90%)	47 (94%)	35 (86%)	0.148 ^Chi^
Coronary flow after PCI: TIMI 3	89 (95%)	46 (92%)	43 (98%)	0.217 ^Chi^
Medication at discharge				
Acetylsalicyl acid	91 (97%)	47 (94%)	44 (100%)	0.099 ^Chi^
P2Y12 inhibitor	90 (96%)	46 (92%)	44 (100%)	0.055 ^Chi^
β-blocker	76 (81%)	39 (78%)	37 (84%)	0.454 ^Chi^
ACE inhibitor/ARB	83 (88%)	45 (90%)	38 (86%)	0.584 ^Chi^
Statin	93 (99%)	49 (98%)	44 (100%)	0.346 ^Chi^
Spironolactone	21 (22%)	17 (34%)	4 (9%)	0.004 ^Chi^
SGLT2 inhibitor	6 (9%)	4 (8%)	2 (11%)	0.739 ^Chi^
Nocturnal ECG Holter parameters				
Heart rate (bpm)	66 [59, 72]	66 [60, 73]	66 [59, 72]	0.590 ^MWU^
PAC/h *	0.6 [0.2, 2.5]	1.2 [0.3, 3.4]	0.3 [0.1, 1.2]	0.017 ^MWU^
SVT ≥ 1 no (%) *	18 (19)	12 (25)	6 (14)	0.186 ^Chi^
PVC/h	0.5 [0.1, 2.7]	0.7 [0.1, 2.4]	0.3 [0.1, 3]	0.440 ^MWU^
NSVT ≥ 1 no (%)	9 (10)	8 (16)	1 (2)	0.024 ^Chi^
Couplet no (%)	18 (19)	11 (22)	7 (16)	0.454 ^Chi^
Bigeminy no (%)	4 (4)	3 (6)	1 (2)	0.374 ^Chi^
Trigeminy no (%)	1 (1)	1 (2)	0 (0)	0.348 ^Chi^

The values are presented as the means ± standard deviations, medians [interquartile ranges], or numbers of patients (%). ACE: angiotensin-converting enzyme; ARB: angiotensin receptor blocker; ECG: electrocardiogram; h: hour; LAD: left anterior descending; NSTEMI: Non-ST elevation myocardial infarction; NSVT: non-sustained ventricular tachycardia; PCI: percutaneous coronary intervention; PAC: premature atrial complex; PVC: premature ventricular complex; SVT: supraventricular tachycardia; TIMI: thrombolysis in myocardial infarction; SDB: sleep-disordered breathing (apnoea-hypopnoea index < 15 events·h^−1^); ^T^ Student’s *t*-test; ^Chi^ Chi-squared test; ^MWU^ Mann–Whitney U test. * One patient of the SDB group was excluded from this analysis due to the presence of atrial fibrillation.

**Table 2:** Nocturnal ECG Holter parameters for the subgroup of patients with SDB randomised to receive ASV treatment in addition to standard care after AMI (ASV n = 13 and control n = 19) with analysable ECG recordings at baseline and at 12-week follow-up.

**Continuous**	**Baseline**	**Week 12**	**Difference**	***p*** **-Value ^a^**
Heart rate (bpm)				
○ ASV (n = 13)	66 [61, 75]	51 [48, 51]	−9 [−17, −7]	0.514 ^MWU^
○ Control (n = 19)	61 [58, 71]	55 [52, 59]	−8 [−18, −3]	
PAC/h				
○ ASV (n = 13)	1.3 [0.2, 35.8]	0.6 [0.1, 4.2]	−0.3 [−11.9, 0.1]	0.645 ^MWU^
○ Control (n = 19)	0.6 [0.1, 2.9]	0.2 [0.0, 0.7]	−0.1 [−1.6, 0.0]	
PVC/h				
○ ASV (n = 13)	0.4 [0.1, 2.3]	0.5 [0.2, 5.9]	0.0 [−1.4, 5.5]	0.604 ^MWU^
○ Control (n = 19)	0.6 [0.1, 7.6]	0.6 [0.1, 10.5]	0.1 [−0.3, 3.5]	
**Dichotomous**	**Baseline**	**Week 12**	**Odds Ratio**	***p*** **-Value ^b^**
SVT ≥ 1 no (%)				
○ ASV (n = 13)	4 (31)	1 (8)	0.7	0.787 ^Chi^
○ Control (n = 19)	2 (11)	2 (11)		
NSVT ≥ 1 no (%)				
○ ASV (n = 13)	2 (15)	0 (0)	0	0.401 ^Chi^
○ Control (n = 19)	3 (16)	1 (5)		
Couplet no (%)				
○ ASV (n = 13)	3 (23)	1 (8)	0.3	0.307 ^Chi^
○ Control (n = 19)	3 (16)	4 (21)		
Bigeminy no (%)				
○ ASV (n = 13)	0 (0)	1 (8)	1.5	0.780 ^Chi^
○ Control (n = 19)	2 (11)	1 (5)		
Trigeminy no (%)				
○ ASV (n = 13)	0 (0)	1 (8)	0.7	0.787 ^Chi^
○ Control (n = 19)	1 (5)	2 (11)		

The values are presented as the medians [interquartile ranges], or numbers of patients (%). AMI: acute myocardial infarction; ASV: adaptive servo ventilation; bpm: beats per minute; ECG: electrocardiogram; NSVT: non-sustained ventricular tachycardia; PAC: premature atrial complex; h: hour; SVT: supraventricular tachycardia; PVC: premature ventricular complex. ^a^ *p*-value for the group comparison of differences derived from Mann–Whitney U test (^MWU^). ^b^ *p*-value for the group comparison at week 12, derived from chi-squared test (^Chi^).

## Data Availability

The study protocol, statistical analysis plan, analytic code and deidentified individual participant data will be made available from 3 months to 5 years following article publication to researchers who provide a methodologically sound proposal. Requests for the format of the proposal and the proposal should be directed to michael.arzt@ukr.de. To gain access, data requestors will need to sign a data access agreement.

## Supplementary Materials

The following supporting information can be downloaded at: https://www.mdpi.com/article/10.3390/jcdd13040157/s1, Figure S1: Study flow chart; Table S1: Demographics, clinical characteristics, and polygraphy findings at baseline for patients with SDB randomly assigned to either the ASV group in addition to standard care of AMI, or the control group.
