# Supplementary material for: Nocturnal Cardiac Arrhythmias in Sleep Apnoea After Acute Myocardial Infarction and the Effect of Adaptive Servo-Ventilation: An Ancillary Study of the TEAM-ASV I Trial
Source: J Cardiovasc Dev Dis. 2026 Apr 2;13(4):157. doi: 10.3390/jcdd13040157 (PMC13116681; doi:10.3390/jcdd13040157)
Supplement: Supplementary file 1 [file jcdd-13-00157-s001.zip › jcdd-4164109-supplementary.pdf]

Supplementary Material

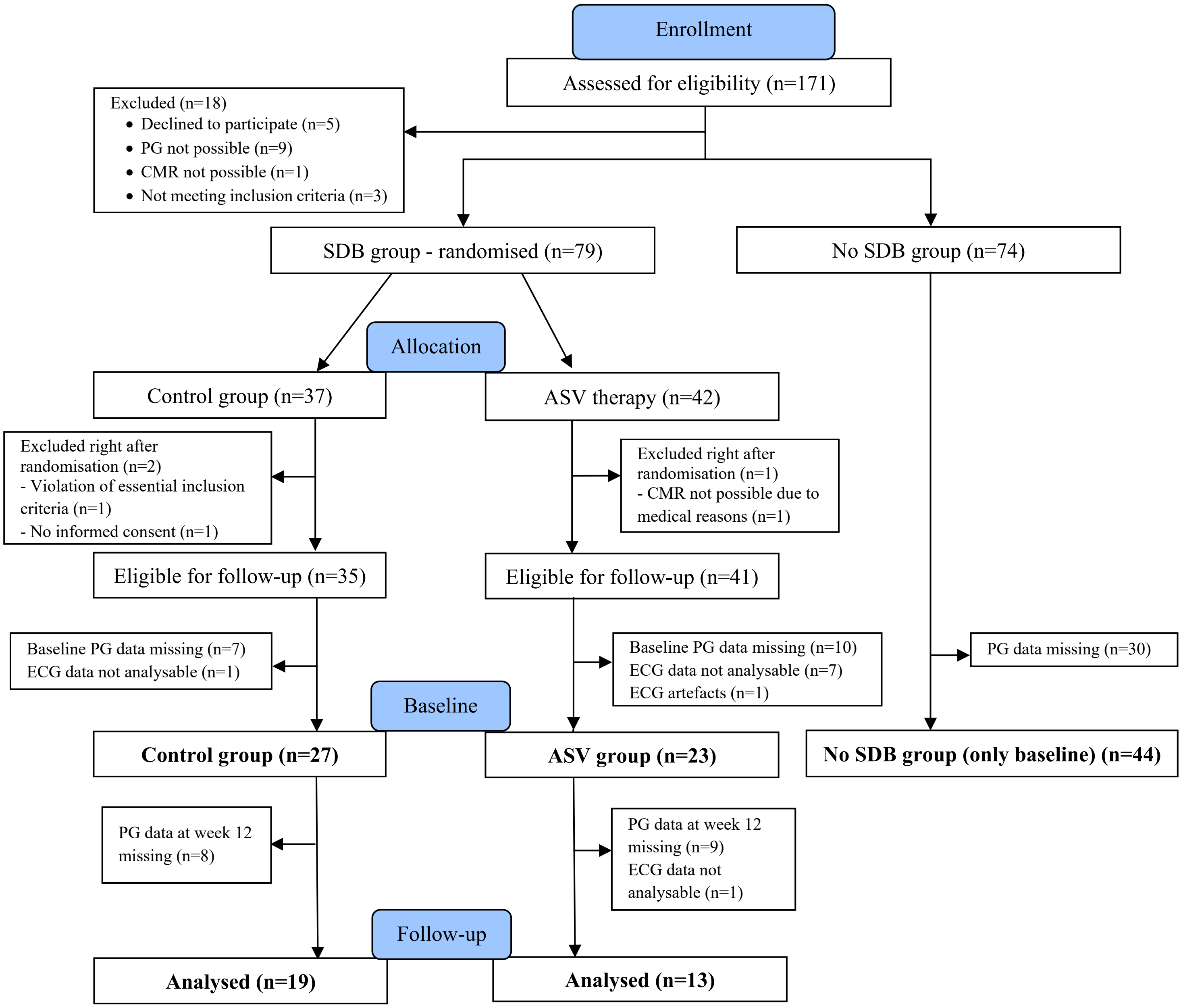

Figure S1: Study flow chart. CMR: cardiac magnetic resonance imaging; PG: polygraphy; SDB: sleep-disordered breathing.

Table S1. Demographics, clinical characteristics, and polygraphy findings at baseline for patients with SDB randomly assigned to either the ASV group in addition to standard care of AMI, or the control group.

	**ASV (n=13)**	**Control (n=19)**	**p-value**
Age, years	62±13	59±8	0.369[T]
Body mass index, kg/m^2	29.8±4.0	30.5±5.0	0.394[T]
Male sex	10 (77%)	18 (95%)	0.135[Chi]
Hypertension	7 (54%)	10 (53%)	0.946[Chi]
Current smoker	4 (31%)	9 (47%)	0.348[Chi]
Diabetes mellitus	1 (8%)	3 (16%)	0.629[F]
Pain-to-balloon time, h	4.9 [3.6-12.6]	4.4 [2.2-8.1]	0.105[MWU]
Left ventricular ejection fraction (%)	50±10	49±12	0.921[T]
NSTEMI	4 (31%)	0 (0%)	0.023[F]
Culprit lesion			
Left main artery	0 (0%)	0 (0%)	
LAD artery	8 (62%)	13 (68%)	0.687[Chi]
Circumflex artery	5 (39%)	3 (16%)	0.146[Chi]
Right coronary artery	0 (0%)	0 (0%)	0.926[Chi]
Coronary flow before PCI: TIMI 0-1	11 (85%)	19 (100%)	0.077[Chi]
Coronary flow after PCI: TIMI 3	13 (100%)	16 (84%)	0.132[Chi]
Medication at discharge			
Acetylsalicyl acid	13 (100%)	17 (90%)	0.227[Chi]
P2Y12 inhibitor	13 (100%)	18 (95%)	0.892[Chi]
β-blocker	11 (85%)	16 (84%)	0.975[Chi]
ACE inhibitor/ARB	12 (92%)	19 (100%)	0.219[Chi]
Statin	13 (100%)	19 (100%)	
Spironolactone	2 (15%)	8 (42%)	0.109[Chi]
SGLT2 inhibitor	2 (15%)	1 (5%)	0.335[Chi]
Polygraphy findings			
Apnoea-hypopnoea index, events/h	41.9±21.7	29.7±12.8	0.086[T]
Apnoea index, events/h	34.4±21.2	20.4±10.4	0.021[T]
Central apnoea-hypopnoea index/apnoea-hypopnoea index, %	39±30	36±30	0.751[T]
Central sleep apnoea	6 (46%)	6 (32%)	0.403[Chi]
Oxygen desaturation index, events/h	22.4±15.0	33.1+23.2	0.160[T]
Minimum oxygen saturation, %	80.9±4.4	80.6±8.0	0.906[T]

Total recording time, h	8.2±0.9	8.0±0.8	0.563^{T}

The values are presented as the means ± standard deviations, medians [interquartile ranges], or numbers of patients (%). ACE: angiotensin-converting enzyme; ARB: angiotensin receptor blocker; ECG: electrocardiogram; h: hour; LAD: left anterior descending; NSTEMI: Non-ST elevation myocardial infarction; NSVT: non-sustained ventricular tachycardia; PCI: percutaneous coronary intervention; PAC: premature atrial complex; PVC: premature ventricular complex; SVT: supraventricular tachycardia; TIMI: thrombolysis in myocardial infarction; SDB: sleep-disordered breathing (apnoea-hypopnoea index <15 events·h−1); ^{T} Student's t-test; ^{Chi} Chi-squared test; ^{MWU} Mann-Whitney U test. *One patient of the SDB group was excluded from this analysis due to the presence of atrial fibrillation.
